# CD47-SIRPα Checkpoint Inhibition Enhances Neutrophil-Mediated Killing of Dinutuximab-Opsonized Neuroblastoma Cells

**DOI:** 10.3390/cancers13174261

**Published:** 2021-08-24

**Authors:** Paula Martínez-Sanz, Arjan J. Hoogendijk, Paul J. J. H. Verkuijlen, Karin Schornagel, Robin van Bruggen, Timo K. van den Berg, Godelieve A. M. Tytgat, Katka Franke, Taco W. Kuijpers, Hanke L. Matlung

**Affiliations:** 1 Sanquin Research and Landsteiner Laboratory, Department of Molecular Hematology, Amsterdam UMC, University of Amsterdam, 1066 CX Amsterdam, The Netherlands; a.hoogendijk@sanquin.nl (A.J.H.); p.verkuijlen@sanquin.nl (P.J.J.H.V.); k.schornagel@sanquin.nl (K.S.); r.vanbruggen@sanquin.nl (R.v.B.); t.k.vandenberg@sanquin.nl (T.K.v.d.B.); katkafranke@gmail.com (K.F.); t.w.kuijpers@amsterdamumc.nl (T.W.K.); 2 Department of Molecular Cell Biology and Immunology, Amsterdam UMC, Vrije Universiteit Amsterdam, 1081 HV Amsterdam, The Netherlands; 3 Princess Maxima Center for Pediatric Oncology, Department of Pediatric Oncology, 3584 CS Utrecht, The Netherlands; g.a.m.tytgat@prinsesmaximacentrum.nl; 4 Department of Pediatric Immunology, Rheumatology and Infectious Diseases, Emma Children’s Hospital, Amsterdam UMC, University of Amsterdam, 1105 AZ Amsterdam, The Netherlands

**Keywords:** neutrophils, neuroblastoma, dinutuximab, antibody-dependent cellular cytotoxicity, antibody therapy, checkpoint blockade, CD47-SIRPα, immunotherapy

## Abstract

**Simple Summary:**

Current immunotherapy for high-risk neuroblastoma patients involves treatment with anti-GD2 antibody dinutuximab, which has significantly improved the survival rate. Still, approximately half of the patients succumb to the tumor; therefore, efforts to improve their prognosis are urgently needed. Since T cell targeting immune checkpoint inhibitors in neuroblastoma are limited due to the low immunogenicity of these tumors, alternative immunotherapeutic approaches should be studied. The therapeutic targeting of the innate immune checkpoint CD47-SIRPα has the ability to enhance antitumor effects of myeloid cells, especially in the presence of cancer-opsonizing antibodies. Given that neutrophil ADCC is a dominant effector mechanism leading to the eradication of dinutuximab-opsonized neuroblastoma cells, we have investigated the therapeutic potential of anti-GD2 antibody in combination with CD47-SIRPα inhibition. We demonstrate here that the capacity of neutrophils to kill dinutuximab-opsonized neuroblastoma cells is controlled by the CD47-SIRPα axis and its disruption promotes their cytotoxic potential even further, significantly improving dinutuximab responsiveness.

**Abstract:**

High-risk neuroblastoma, especially after recurrence, still has a very low survival rate. Immune checkpoint inhibitors targeting T cells have shown remarkable clinical efficacy in adult solid tumors, but their effects in pediatric cancers have been limited so far. On the other hand, targeting myeloid immune checkpoints, such as CD47-SIPRα, provide the opportunity to enhance antitumor effects of myeloid cells, including that of neutrophils, especially in the presence of cancer-opsonizing antibodies. Disialoganglioside (GD2)-expressing neuroblastoma cells targeted with anti-GD2 antibody dinutuximab are in part eradicated by neutrophils, as they recognize and bind the antibody targeted tumor cells through their Fc receptors. Therapeutic targeting of the innate immune checkpoint CD47-SIRPα has been shown to promote the potential of neutrophils as cytotoxic cells in different solid tumor indications using different cancer-targeting antibodies. Here, we demonstrate that the capacity of neutrophils to kill dinutuximab-opsonized neuroblastoma cells is also controlled by the CD47-SIRPα axis and can be further enhanced by antagonizing CD47-SIRPα interactions. In particular, CD47-SIRPa checkpoint inhibition enhanced neutrophil-mediated ADCC of dinutuximab-opsonized adrenergic neuroblastoma cells, whereas mesenchymal neuroblastoma cells may evade immune recognition by a reduction of GD2 expression. These findings provide a rational basis for targeting CD47-SIRPα interactions to potentiate dinutuximab responsiveness in neuroblastomas with adrenergic phenotype.

## 1. Introduction

Immune checkpoint inhibitors of the adaptive immune system have in the last decades revolutionized the treatment landscape of cancer by demonstrating unprecedented success across a wide spectrum of adult advanced cancers [1]. Despite the progress seen with checkpoint blockade in adults, the use of such approaches in pediatric cancers has to date failed to show meaningful clinical efficacy [2,3]. One major factor behind the pediatric tumor resistance to immune checkpoint immunotherapy is considered to be the low mutation rate that these tumors present [4,5,6]. This results in an important scarcity of neoantigens that can be recognized by T cells, giving rise to a lack of T cell-containing or so-called ‘cold tumors’. Unfortunately, patients with such tumors appear unable to benefit from T cell-directed checkpoint blockade therapies. Another consideration for immunotherapy resistance is the highly sophisticated immunosuppressive tumor microenvironment found in most pediatric malignancies [7,8]. The presence of M2 (pro-tumoral) macrophages and a dense stroma packed with fibroblasts is believed to prevent effective adaptive immune responses [2].

Neuroblastoma, an aggressive and bulky cancer affecting very young children, is one example of a pediatric tumor in which the above-mentioned features are inherently present; hence, little has been accomplished regarding the application of adaptive checkpoint blockade immunotherapies in this tumor type. There are, however, effector immune cells other than T cells that can efficiently combat cancer and lead to the eradication of the tumor cells. Neutrophils are the most abundant leukocytes present in human blood and compelling evidence has put them in the spotlight as cells with significant antitumor capacities [9,10,11]. Among their immune-mediated effects is induction of tumor cell death of antibody opsonized cancer cells, a process known as antibody-dependent cellular cytotoxicity (ADCC) [12,13]. In neuroblastoma, the standard of care for high-risk patients involves antibody therapy with the anti-disialoganglioside (GD2) antibody dinutuximab, which has greatly increased the survival of patients since its implementation in the treatment protocol [14,15]. Among others, neutrophil ADCC has been recognized as an important effector mechanism contributing to the eradication of these dinutuximab-opsonized neuroblastoma cells [16]. Evidence for the relevant role of neutrophils in this cancer type comes from the favorable correlation with clinical outcome found after anti-GD2 immunotherapy with a specific polymorphic variant of *FCGR2A* [17]. This receptor, present exclusively on myeloid cells, represents the predominant activating FcγR present on neutrophils, and it has been demonstrated to be the principal mediator of neutrophil ADCC through recognition of the IgG tail of cancer-targeting therapeutic antibodies directed against other solid cancer cells [18,19,20]. Furthermore, the overall clinical response of neuroblastoma patients has been found to be further improved by the addition of granulocyte-macrophage colony-stimulating factor (GM-CSF), acting on myeloid cells including neutrophils, to the anti-GD2 treatment regime [14,21,22,23,24]. Overall, from the above-mentioned studies and a variety of others, it has further become apparent that neutrophils can be stimulated by cytokines such as GM-CSF or granulocyte colony-stimulating factor (G-CSF), given either alone or in combination with interferon-gamma (IFNγ), in order to improve their in vitro IgG-mediated cytotoxicity [25,26,27,28,29].

Neutrophils, as well as T cells, are endowed with inhibitory receptors so as to suppress their activity when necessary, which turns them into potential therapeutic targets for checkpoint blockade therapy [30,31]. Signal regulatory protein alpha (SIRPα) is one well-established example of an immunoreceptor expressed on neutrophils that can be successfully targeted for checkpoint-blockade. Its ligand, CD47, a molecule present on normal cells that generally acts as a ‘don’t eat me signal’, is often overexpressed by tumor cells, leading to an evasion of tumor cell recognition and hampered elimination by the immune system [32]. We and others have shown that CD47-SIRPα interactions negatively regulate antibody-mediated cytotoxicity by neutrophils both in vitro and in vivo for a number of cancers (i.e., Her2^+^-breast cancer, EGFR^+^-carcinoma), and that a blockade of the interaction substantially potentiates the cytotoxic capabilities of these effector cells [33,34,35]. In a clinical setting, several approaches to block CD47-SIRPα axis are already in clinical development for multiple cancer indications [36,37,38]. The involvement of the CD47-SIRPα checkpoint in neuroblastoma has, however, not been thoroughly investigated yet. Here, we examined whether an inhibition of CD47-SIRPα axis, by either a genetic disruption or by using an antagonistic agent for SIRPα, allows neutrophils to more efficiently kill dinutuximab-opsonized neuroblastoma cells in vitro. By testing cells of either an adrenergic or mesenchymal phenotype, the two divergent cellular phenotypes responsible for a large part of the tumor heterogeneity found in neuroblastoma, we further characterized the involvement of CD47-SIRPα checkpoint in this cancer type. Overall, this provides a rational basis for the targeting of CD47-SIRPα interactions to improve the clinical response to anti-GD2 therapy in children suffering from neuroblastoma.

## 2. Materials and Methods

### 2.1. Neutrophil Gene Signature Enrichment in Neuroblastoma Samples

Neuroblastoma stage-specific regulated transcript listings were obtained from Zhang et al. [39]. Regulated transcripts were collapsed to SYMBOL identifiers. We defined a neutrophil gene signature based on all transcripts that were upregulated in a dataset of differentiating primary neutrophils [40]. These neutrophil transcripts were collapsed to SYMBOL identifiers and merged with the neuroblastoma regulated transcript list.

### 2.2. mRNA Data Analysis for CD47 Expression

mRNA sequencing data on expression levels for *CD47* from healthy tissue and neuroblastoma tumors collected from Genotype-Tissue Expression Project (GTEx) and the Therapeutically Applicable Research to Generate Effective Treatment Program (TARGET) studies, respectively, were downloaded as log2 values from the Xena Functional Genomics Explorer (https://xenabrowser.net/, accessed on 21 February 2021) under the query “TCGA TARGET GTEx”. GTEx healthy tissue samples were filtered in for adrenal gland, while TARGET tumor samples were filtered in for neuroblastoma.

Microarray sequencing data regarding *CD47* mRNA expression levels in the different neuroblastoma disease stages were obtained from the Zhang et al. dataset with Gene Expression Omnibus (GEO) accession number GSE49710 [39]. Other *CD47* expression profiles used in this study are available from GEO: neuroblastoma cell line panel (GSE28019) and isogenic neuroblastoma cell line pairs of adrenergic or mesenchymal phenotypes (GSE90803). All gene expression analyses were performed in the R2 genomics analysis and visualization platform (http://r2.amc.nl, accessed on 22 March 2021). Where applicable, normalization for expression was based on the expression of *GUSB* and was defined as log2 *CD47*-log2 *GUSB*. Supplemental data on *CD47* expression of other databases analyzed in the present study can be accessed from GEO or R2 browser with the following identifiers: adrenal gland (various: GSE3527, GSE7307, GSE8514) and neuroblastoma (GSE49710, GSE16476, GSE14880, GSE16237, GSE13136).

### 2.3. Neutrophil Isolation and Stimulation

Neutrophils from heparinized peripheral blood were isolated as previously described by density gradient centrifugation with isotonic Percoll (GE Healthcare, Chicago, IL, USA) and erythrocyte lysis with ice cold hypotonic ammonium chloride solution [41]. Neutrophils were used either directly after isolation (unstimulated) or were stimulated for 30 min or overnight at 37 °C and 5% CO_2_ with recombinant human GM-CSF (10 ng/mL; Peprotech, Cranbury, NJ, USA), clinical grade G-CSF (10 ng/mL; Neupogen, Amgen, Thousand Oaks, CA, USA), or a combination of clinical grade G-CSF (10 ng/mL) and recombinant human IFNγ (50 ng/mL; Peprotech). After overnight incubation, the percentage of apoptotic cells was determined using Annexin V staining (BD Biosciences, Franklin Lakes, NJ, USA) to correct for the number of viable neutrophils prior to any experiments. All human blood samples were obtained and used according to the declaration of Helsinki 1964.

### 2.4. Cell Culture

The human neuroblastoma cell lines NMB, LAN-1, and IMR-32 were obtained in 2018 from the Leibniz Institute DSMZ, Germany. These cells were routinely cultured at 37 °C and 5% CO_2_ and maintained in Iscove’s modified Dulbecco’s medium (IMDM; Thermo Fisher Scientific, Waltham, MA, USA) supplemented with 20% of heat-inactivated fetal calf serum (FCS), 2 mM L-glutamine, 100 units/mL penicillin, and 100 μg/mL streptomycin. The human neuroblastoma cell lines SHEP-2 and SK-N-AS (kindly provided by the department of Oncogenomics, Amsterdam UMC, Amsterdam, The Netherlands) were routinely cultured at 37 °C and 5% CO_2_ and maintained in Dulbecco’s modified Eagle medium (DMEM; Thermo Fisher Scientific) supplemented with 20% of FCS, 0.1 mM non-essential amino acids, 2 mM L-glutamine, 100 units/mL penicillin, and 100 μg/mL streptomycin.

The primary patient-derived neuroblastoma spheroid lines AMC691T and AMC691B (hereafter 691T and 691B) were derived from either the primary tumor site (T) or a bone marrow metastasis (B) of patient 691 [42]. 691T and 691B cells were cultured and maintained in DMEM (Thermo Fisher Scientific) with low glucose and sodium pyruvate (Thermo Fisher Scientific) supplemented with 25% Ham’s F12 nutrient mixture (Thermo Fisher Scientific), B-27 supplement minus vitamin A (50X; Thermo Fisher Scientific), N-2 supplement (100X; Thermo Fisher Scientific), 20 ng/mL animal-free human epidermal growth factor (Peprotech), 40 ng/mL human basic fibroblast growth factor (Peprotech), 200 ng/mL human insulin-like growth factor (Peprotech), 10 ng/mL human platelet-derived growth factor-AA (Peprotech), 10 ng/mL human platelet-derived growth factor-BB (Peprotech), 100 units/mL penicillin, and 100 μg/mL streptomycin.

All cells were kept in culture for up to 3 months and were routinely tested for potential mycoplasma infection using polymerase chain reaction. 

### 2.5. Generation of Genetically Modified Cells

CD47 was knocked out from the neuroblastoma cell lines by lentiviral plasmid pLentiCrispRv2 transduction (Addgene, Watertown, MA, USA) containing gRNA against the gene of interest. Knockout cells for CD47 were obtained when using either gRNA 5′ CCAGCAACAGCGCCGCTACC 3′ (hereafter CD47 KO1) or gRNA 5′ CAGCAACAGCGCCGCTACCA 3′ (hereafter CD47 KO2). Tumor cells expressing scrambled gRNA were used as a control for transduction (scrambled: 5′ GCACTACCAGAGCTAACTCA 3′). Lentivirus was grown by transient transfection of HEK293T cells. Virussup was harvested on day 2 and 3 after transfection, filtered through 0.45 µM, and added to the target cells. Transduced cells were selected with 1–2 μg/mL Purocymin (Invivogen, San Diego, CA, USA) and were kept in Puromycin selection until flow cytometry sorting on BD FACSAria^™^ III Cell Sorter (BD Biosciences). The transduction resulted in 60% to 80% of cells with no CD47 expression, and CD47 KO cells were collected and further expanded in culture. Knockout of CD47 on the different cell lines was routinely verified by flow cytometry.

### 2.6. Flow Cytometry Staining

For GD2 detection on target cells, the human anti-GD2 antibody dinutuximab (Unituxin, Ch14.18; United Therapeutics, Silver Spring, MD, USA) was previously conjugated to a 633 dye with Lightning-Link^™^ Atto 633 kit (Innova Biosciences Ltd., Cambridge, UK) according to manufacturers’ instructions. After conjugation, 10 µg/mL of the directly labeled dinutuximab was used to quantify GD2 expression by flow cytometry. To detect CD47 on target cells, 10 μg/mL anti-human CD47 (clone B6H12; own hybridoma) and Alexa Fluor 633 F(ab’)_2_ antibody (Thermo Fisher Scientific) were used for primary and secondary staining, respectively. Cell viability of target cells was determined using Hoechst 33,342 solution (Thermo Fisher Scientific). SIRPα expression on neutrophils was detected with 10 μg/mL anti-human SIRPα (clone 12C4; own hybridoma) and a subsequent incubation with Alexa Fluor 488 antibody (Thermo Fisher Scientific) for secondary staining, or with a FITC-labeled antibody on SHEP-2 and SK-N-AS tumor cells. Where needed, isotypes and secondary antibody controls were used to correct for any potential background. Fluorescence was measured on BD FACSCanto ^™^ II flow cytometer (BD Biosciences) and data were analyzed with FlowJo software (version 10.6.1, Becton Dickinson, Ashland, OR, USA).

### 2.7. ADCC

Target cells (1 × 10^6^) were labeled in their culture medium for 90 min at 37 °C with 100 µCi ^51^Cr (PerkinElmer, Waltham, MA, USA) and finally diluted to 0.1 × 10^6^ cells/mL after several washing steps. Neutrophils were either left untreated or were pre-incubated with 10 µg/mL anti-SIRPα at room temperature for the indicated conditions. Co-incubation of target and effector cells was carried out at a target:effector (T:E) ratio of 1:50 (i.e., 5000:250,000 cells), unless specified otherwise, for 4 h at 37 °C and 5% CO_2_ in the absence or presence of 0.5 µg/mL dinutuximab. Spontaneous and maximum ^51^Cr release were determined by incubating the target cells without effector cells and by treating them with a 0.1% triton X-100 (Sigma Aldrich, St. Louis, MO, USA), respectively. After incubation, supernatant was harvested and analyzed for radioactivity in a Wallac Wizard gamma counter or a MicroBeta^2^ plate reader (PerkinElmer). The percentage of cytotoxicity was calculated as: [(experimental counts per minute (CPM)-spontaneous CPM)/(maximum CPM-spontaneous CPM)] × 100%. All conditions were performed in duplo or triplicate.

### 2.8. Statistical Analysis

Gene overrepresentation for neutrophil gene signature enrichment in neuroblastoma samples was determined using Fisher exact tests. Where applicable, *p*-values were adjusted using Benjamini–Hochberg multiple test correction. Statistical differences between groups were evaluated by one- or two-way ANOVA, or by student’s *t*-test using GraphPad Prism version 8. Where indicated, correction for multiple comparisons using either Sidak’s or Tukey’s test was performed. Data were considered significant when *p* < 0.05. The results are presented as the mean ± standard error of the mean.

## 3. Results

### 3.1. Neuroblastoma Tumors Contain Neutrophil mRNA Signatures and Upregulate CD47 Expression

**Figure 1:**
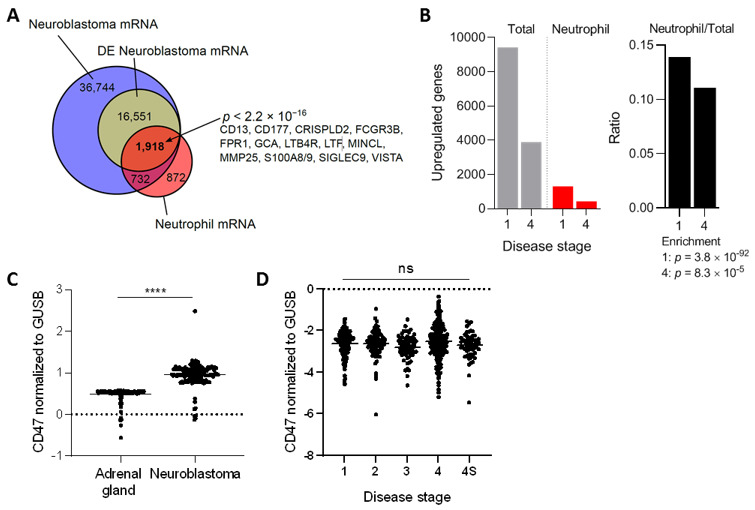
(**A**) Venn diagram showing overlap between neuroblastoma (Zhang et al. [39]) and neutrophil (Grassi et al. [40]) regulated transcripts. Overrepresentation was determined with a Fisher exact test. DE, differentially expressed. (**B**) Proportion of neutrophil associated genes in neuroblastoma stratified according to disease stages 1 and 4 (left panel: absolute numbers, right panel: ratios). Enrichment determined with Fisher exact tests. (**C**) Normalized *CD47* mRNA expression levels in healthy adrenal gland and neuroblastoma tumors. Adrenal gland: *n* = 127, neuroblastoma: *n* = 162. Statistical significance was tested with unpaired *t*-test; **** *p* < 0.0001. (**D**) Normalized *CD47* mRNA expression levels in neuroblastoma patients stratified by disease stage. Stage 1: *n* = 121, stage 2: *n* = 78, stage 3: *n* = 63, stage 4: *n* = 183, stage 4S: *n* = 53. Statistics were performed by one-way ANOVA; ns, not significant.

To further support the idea that neutrophils are relevant players in neuroblastoma tumors, we first investigated the presence of neutrophil mRNA markers in a dataset based on almost 500 biopsies of primary neuroblastomas [39]. From the 55.945 transcripts found in this malignancy, 18,469 were found to be differentially expressed. The differentially expressed neuroblastoma mRNA transcripts were compared with a neutrophil mRNA gene signature extracted from Grassi et al. [40]. This analysis revealed that 1.918 of the 18.469 differentially expressed neuroblastoma transcripts were neutrophil-related, which included highly specific neutrophil markers such as *FCGR3B*, *FPR1, S100A8/9*, and *SIGLEC9*, among others (Figure 1A). Furthermore, the ratio of upregulated neutrophil mRNA signature versus total mRNA indicated a significant influx of neutrophils in neuroblastoma tumors of either disease stage 1 (very low risk) or 4 (high risk), as established by the International Neuroblastoma Staging System [43] (Figure 1B). Altogether, these data insinuate that both early as well as advanced stage neuroblastoma tumors contain neutrophil mRNA signatures.

To assess the relevance of CD47-SIRPα signaling in neuroblastoma, we first examined the gene expression levels of CD47 in this tumor type and compared it to the levels in the respective healthy tissue, being the adrenal gland in our case [44,45]. To do so, mRNA-sequencing data from the publicly available GTEx study was used to extract the data from healthy tissue, which was filtered in for adrenal gland tissue. Meanwhile, the TARGET study, specialized in genomic data of pediatric cancers, was used to obtain the respective mRNA data of neuroblastoma samples. We found that human neuroblastoma tumors expressed significantly higher levels of the immune checkpoint molecule CD47 relative to normal adrenal gland tissue (Figure 1C), suggesting a pronounced ~twofold upregulation on mRNA level of the molecule. To check whether this finding was not an isolated phenomenon for this particular database, four other neuroblastoma datasets (GSE49710, GSE16476, GSE14880, GSE16237, GSE13136) were examined in which a significant CD47 overexpression was found in three out of the four studies when compared to the adrenal gland values of a different dataset (GSE3527, GSE7307, GSE8514; Figure S1). In addition, we investigated CD47 mRNA expression levels in neuroblastoma stages 1, 2, 3, 4, and 4S in the cohort of Zhang et al. [39]. After disease stage stratification we found that CD47 expression remained high and was unaltered over all stages (Figure 1D).

### 3.2. CD47-SIRPα Disruption Potentiates Neutrophil-Dependent Antitumor Activity towards Neuroblastoma Cells

**Figure 2:**
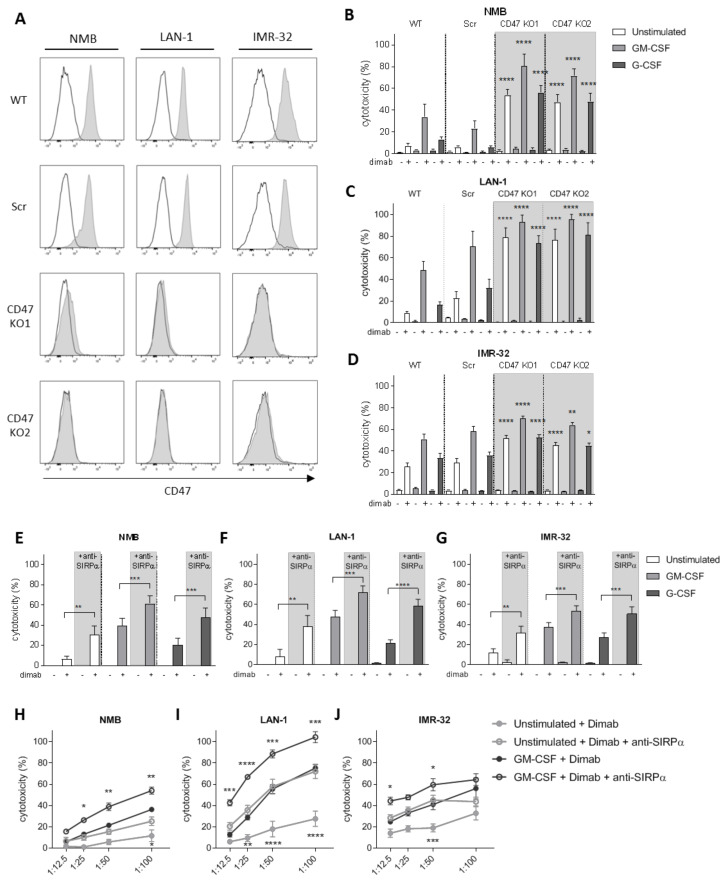
(**A**) Representative histograms depicting CD47 surface expression (*n* = 3) as analyzed by flow cytometry on (from left to right) NMB, LAN-1, and IMR-32 control cells (top two rows) and their respective CD47 KO variants (bottom two rows). Secondary antibody controls are represented in white. (**B**–**D**) ADCC of control (WT and Scr, no background) and CD47 KO (CD47 KO1 and CD47 KO2, grey background) NMB (**B**), LAN-1 (**C**), and IMR-32 (**D**) cells opsonized with (+) or without (−) dinutuximab (dimab) by unstimulated neutrophils (white bars) or stimulated with GM-CSF (light grey bars) or G-CSF (dark grey bars). *n* = 6, of 3 individual experiments. Statistics were performed by one-way ANOVA with Sidak correction for multiple comparisons. (**E**–**G**) ADCC of NMB (**E**), LAN-1 (**F**), and IMR-32 (**G**) cells opsonized with (+) or without (−) dinutuximab (dimab) by unstimulated neutrophils (white bars) or stimulated with GM-CSF (light grey bars) or G-CSF (dark grey bars) in the absence (no background) or presence (grey background) of SIRPα blocking agent. *n* = 6–14, of 7 independent experiments. Statistical analysis was assessed with by a paired *t*-test. (**H**–**J**) ADCC of dinutuximab-opsonized NMB (**H**), LAN-1 (**I**), and IMR-32 (**J**) cells by unstimulated neutrophils (light grey circles) or stimulated with GM-CSF (dark grey circles) in the absence (filled circles) or presence (empty circles) of SIRPα blocking agent at different T:E ratios ranging from 1:12.5 to 1:100. *n* = 5, of 4 individual experiments. Statistical differences were tested with two-way ANOVA with Tukey’s post hoc test; * *p* < 0.05; ** *p* < 0.01; *** *p* < 0.001; **** *p* < 0.0001. ADCC, antibody-dependent cellular cytotoxicity. WT, wildtype. Scr, scrambled.

To investigate the role of CD47-SIRPα as checkpoint in neutrophil-mediated ADCC in neuroblastoma, we verified CD47 expression in the GD2-positive neuroblastoma cell lines NMB, LAN-1, and IMR-32 (Figures 2A and S2A). To genetically disrupt CD47-SIRPα interaction, CD47 expression was deleted on all cell lines by CrispR/Cas9 with two different guide RNAs using lentiviral transduction. This resulted in full CD47 knockouts (KO; Figure 2A) that did not interfere with GD2 expression on any of the cell lines (Figure S2A). Co-cultured dinutuximab-opsonized CD47 KO cells with neutrophils expressing high levels of SIRPα (Figure S3A,B) were more readily killed as compared with wild-type or scrambled cells (used as control for transduction). This resulted in significantly higher levels of neutrophil-mediated cytotoxicity regardless of the stimulus used (Figure 2B–D). Importantly, the most compelling enhancing effect was seen for unstimulated neutrophils, for which unmodified neuroblastoma cells as targets barely resulted in 5–20% of killing, whereas for the CD47 KO cell lines, the neutrophil-mediated cytotoxicity levels were enhanced up to 50–80%. This enhancement in tumor cell killing of CD47 KO cell lines happened similarly upon a longer stimulation overnight of the neutrophils with either GM-CSF or G-CSF cytokines (Figure S2B–D). Of note, as for the genetically unmodified target cells, no killing of the CD47 deleted cells occurred in the absence of the therapeutic antibody dinutuximab, demonstrating that CD47–SIRPα interactions only control antibody-dependent mechanisms of neuroblastoma killing by neutrophils.

Since neutrophils are considered promising effector cells for anti-SIRPα antibody therapy as these cells express high levels of this inhibitory receptor both at a basal state (unstimulated) and upon stimulation with growth factors and cytokines (Figure S3A,B), we next evaluated the effect of directly blocking SIRPα in vitro. The therapeutic activity of the antagonistic antibody against SIRPα was first tested either alone or in combination with the tumor-opsonizing antibody dinutuximab on the above-mentioned GD2-positive neuroblastoma cell lines NMB, LAN-1, and IMR-32 (wild types). Blockade of SIRPα resulted in a significantly augmented neutrophil-mediated ADCC of all three cell lines when co-cultured with either unstimulated or stimulated neutrophils with GM-CSF alone, G-CSF alone, or G-CSF in combination with IFNγ (Figures 2E–G and S2E–G). In line with our data on CD47 KO neuroblastoma cells, we found the SIRPα blocking agent alone did not induce neutrophil-mediated cytotoxicity of tumor cells unopsonized with dinituximab. To test the effect of decreasing numbers of neutrophils available, we investigated the cytotoxic capabilities of anti-SIRPα treated neutrophils by reducing their numbers. We found that even at relatively low T:E ratios, i.e., 1:12.5 or 1:25, the therapeutic activity of the SIRPα blocking antibody was still detectable, which can be especially appreciated for LAN-1 and IMR-32 target cells (Figures 2H–J and S2H–J). Particularly, the condition of unstimulated neutrophils following anti-SIRPα treatment resulted in cytotoxicity levels as high as those induced by GM-CSF stimulated neutrophils in the absence of SIRPα blocking antibody.

### 3.3. Tumor Cell Opsonization Determines Anti-SIRPα Treatment Efficacy

**Figure 3:**
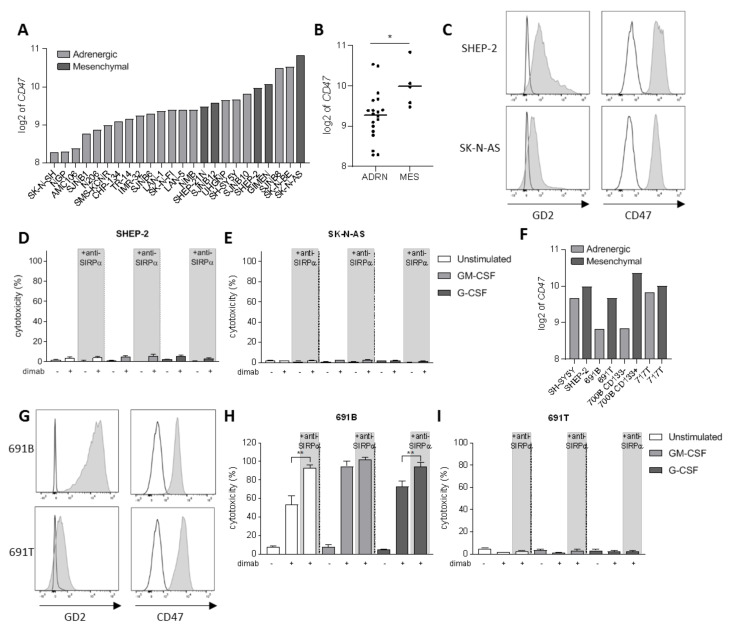
(**A**) CD47 mRNA expression levels (expressed as log2) on a panel of 24 neuroblastoma cell lines. Depicted in light grey are the cell lines of adrenergic phenotype while dark grey bars represent mesenchymal cell lines. (**B**) Scatterplot showing the pooled CD47 mRNA expression (expressed as log2) of the panel of 24 neuroblastoma cell lines divided by phenotype: adrenergic (*n* = 19) and mesenchymal (*n* = 5). Statistical significance was assessed with unpaired *t*-test; * *p* < 0.05. (**C**) Representative histograms depicting GD2 (left) and CD47 (right) surface expression (*n* = 3) as analyzed by flow cytometry on SHEP-2 (top) and SK-N-AS (bottom) cells. Isotype and secondary antibody controls are represented in white. (**D**,**E**) ADCC of SHEP-2 (**D**) and SK-N-AS (**E**) cells opsonized with (+) or without (−) dinutuximab (dimab) by unstimulated neutrophils (white bars) or stimulated with GM-CSF (light grey bars) or G-CSF (dark grey bars). *n* = 6, of 3 individual experiments. Statistical significance was tested with a paired *t*-test. (**F**) CD47 mRNA expression (expressed as log2) on a panel of four isogenic neuroblastoma cell line pairs with opposite phenotype: adrenergic (light grey) and mesenchymal (dark grey). (**G**) Representative histograms showing GD2 (left) and CD47 (right) surface expression (*n* = 2) as analyzed by flow cytometry on 691B (top) and 691T (bottom) cells. Isotype and secondary antibody controls are represented in white. (**H**,**I**) ADCC of primary patient-derived 691B (**H**) and 691T (**I**) cells opsonized with (+) or without (−) dinutuximab (dimab) by unstimulated neutrophils (white bars) or stimulated with GM-CSF (light grey bars) or G-CSF (dark grey bars) *n* = 4–6, of 3 individual experiments. Statistical significance was tested with a paired *t*-test; ** *p* < 0.01. ADRN, adrenergic. MES, mesenchymal.

Another level of complexity in neuroblastoma tumors being responsible for the intratumoral heterogeneity among patients is the existence of two divergent cellular phenotypes with distinct gene expression profiles: the adrenergic and mesenchymal phenotypes [46,47]. It is now widely established that committed adrenergic neuroblastoma cells can switch their fate and interconvert into undifferentiated mesenchymal cells, which are known to be enriched in post-therapy and relapsing tumors as a result of epithelial-to-mesenchymal transition processes [46]. Therefore, we sought to investigate the involvement of CD47-SIRPα axis in neuroblastoma cells of mesenchymal phenotype as well. First, we examined CD47 mRNA expression on a panel of 24 neuroblastoma cell lines from tissue banks which included cells of both phenotypes (Figure 3A). We found a significantly higher expression of CD47 on cell lines with a mesenchymal phenotype compared to the adrenergic cells (Figure 3B). From these, we focused on two of the mesenchymal cell lines: SHEP-2 and SK-N-AS, which were characterized for GD2 and CD47 expression by flow cytometry. SHEP-2 and SK-N-AS cells express relatively low levels of GD2 antigen compared to one of the earlier mentioned adrenergic cell lines, LAN-1 (Figures 3C and S4A). Both cell lines were confirmed to express CD47 at high levels also at protein level (Figure 3C), and, in line with the mRNA expression dataset (Figure 3A), were found to express higher CD47 levels as compared to LAN-1 (Figure S4B). Despite the high expression of the SIRPα ligand on the surface of SHEP-2 and SK-N-AS cell lines, a SIRPα block on either unstimulated or stimulated neutrophils in an ADCC assay had no effect on the cytotoxicity of these cells (Figure 3D,E). This suggests it is not the inhibitory signal provided by SIRPα binding to CD47 on these tumor cells that hampers neutrophil-mediated ADCC, but rather an insufficient tumor cell opsonization due to low GD2 expression is limiting the full cytotoxic capacity of neutrophils towards dinutuximab-opsonized SHEP-2 and SK-N-AS cells. Although SHEP-2 and SK-N-AS cells were shown to express relatively low levels of SIRPα on their surface (Figure S3C,D), no neutrophil-cytotoxicity effect was detected in the conditions with anti-SIRPα treatment only, suggesting that the level of opsonization that the anti-SIRPα antibodies could possibly cause on the tumor cells is not sufficient to trigger neutrophil killing by itself.

To better evaluate the involvement of CD47-SIRPα axis in the two divergent phenotypes that can be found in neuroblastoma, we examined CD47 expression in four isogenic neuroblastoma cell line pairs with opposite phenotypes that were isolated from individual patients [48]. Again, a trend for higher CD47 mRNA expression was found for the cells of mesenchymal phenotype, as compared to their respective adrenergic pair (Figure 3F). Of these, we further characterized the isogenic pair from patient 691—691T cells were derived from the primary tumor site of a neuroblastoma patient while 691B cells were isolated from the bone marrow metastasis of the same patient [42]—for the markers of interest by flow cytometry. CD47 expression could be detected on the surface of both counterparts (Figure 3G), and, despite not showing statistical significance, its expression seemed higher for the mesenchymal 691T cells as compared to the adrenergic 691B cells (Figure S4B), correlating with the findings at mRNA level (Figure 3F). The 691B cell line of adrenergic phenotype expressed GD2 in the same order of magnitude compared to LAN-1, while the mesenchymal 691T cells lost most of the expression of the ganglioside on the surface membrane, similar to SK-N-AS (Figures 3G and S4A). Next, we assessed the ability of neutrophils to kill the two primary patient-derived tumor cell lines 691B and 691T. Anti-SIRPα treatment of neutrophils further enhanced the killing of GD2-positive adrenergic 691B cells, reaching cytotoxicity levels up to 100%, even by unstimulated neutrophils (Figures 3H and S4C). Conversely, SIRPα blockade did not induce any neutrophil-mediated killing of GD2-low expressing mesenchymal 691T cells (Figures 3I and S4D). Altogether, these results suggest that SIRPα blockade therapy may only be applicable and of benefit when the tumor antigen GD2 is present on the surface of neuroblastoma cells in sufficient amounts and hence ADCC can be triggered upon antibody therapy with dinutuximab.

## 4. Discussion

In recent years, multiple immunotherapeutic approaches have demonstrated promise in the field of pediatric oncology. One undeniable example of this is the use of antibody therapy targeting GD2 with dinutuximab in neuroblastoma. The implementation of dinutuximab into the standard of care for neuroblastoma has significantly increased the 5-year survival rate of high-risk patients from roughly 20% to 50% [49]. Despite the encouraging results of dinutuximab treatment, the prognosis of high-risk neuroblastoma patients remains poor; therefore, intense attempts are currently being made to identify novel immunotherapeutic approaches for the treatment of neuroblastoma. The reduced infiltration and activity of lymphocytes in this low immunogenic tumor, as well as in other pediatric tumors [2,8,50], limits, for now, the application of T cell targeting immune checkpoint inhibitors; therefore, the efforts of researchers are directed at the exploitation of other powerful immune modalities involving, for instance, the innate immune system.

In the present study, we have investigated the role of the CD47-SIRPα innate immune checkpoint in the context of antibody therapy with dinutuximab in neuroblastoma. First, we provided evidence of neutrophil infiltration in neuroblastoma tumors, as well as an upregulation of CD47 molecule throughout all disease stages relative to the levels found in the adrenal gland. These findings were used as a basis to study the involvement of CD47-SIRPα interactions in the neutrophil-mediated cytotoxicity of neuroblastoma cells. We found CD47-SIRPα interactions between neutrophil and tumor cells to limit the neutrophil’s capability of inducing antibody-mediated cytotoxicity in vitro. This was shown by either genetically deleting CD47 molecules from the surface of several neuroblastoma cells or by using a blocking antibody for SIRPα. Just as found for other cancer types [18,33,34], we demonstrated how a disruption of the interaction potentiated the killing capabilities of neutrophils resulting in higher cytotoxicity towards the dinutuximab-opsonized target cells.

From a therapeutic point of view, it seems beneficial to focus on the targeting of SIRPα with a blocking agent, given its more restricted expression on myeloid cells as compared to the ubiquitous expression of CD47 [36]. Nonetheless, clinical trials that are currently being explored with antibodies targeting the CD47-SIRPα axis from the CD47 side in combination with tumor-specific monoclonal antibody therapy have shown minimal to moderate toxicity effects [36,37,38]. More importantly, the success rates of these clinical trials for adult cancers, together with the pre-clinical findings described in the present study, clearly support the clinical application of such a therapeutic approach for neuroblastoma patients in the near future. Furthermore, the experiments where lower neutrophil T:E ratios were used demonstrate the strength of anti-SIRPα treatment as it still significantly enhanced the cytotoxicity levels in the presence of low neutrophil counts.

In the absence of opsonizing dinituximab, we found no enhancing effects of CD47-SIRPα blockade, suggesting that CD47-SIRPα blockers may primarily be useful in combination with dinutuximab, and obviously when GD2 is present. This could be a potential drawback for patients with GD2-negative/low neuroblastoma variants that will not benefit from anti-GD2 immunotherapy. Despite the fact that the loss of GD2 antigen following monoclonal antibody therapy has been described as a rare phenomenon [51], it has been detected in a number of cases [52]. The prevalence of this event could also be appreciated in the present study, wherein we found neuroblastoma cells of mesenchymal phenotype to have lost some or all of the expression of the ganglioside on their surface membrane. To date, the exact mechanism behind GD2 loss is not fully understood, but the results from a recent study by Terzic et al. suggest that resistance to anti-GD2 immunotherapy may be due to selection, i.e., the presence of GD2-negative/low cells in primary tumors that may preferentially grow out during therapy [53].

The combination of GD2 loss (antigen-negative/low clones), perhaps together with the overexpression of CD47, may constitute an immune escape mechanism that tumors use in their favor. This can ultimately lead to clinical resistance or recurrence, a mechanism suggested by our findings, and which is in particular present in the mesenchymal phenotype. Therefore, alternative immunotherapeutic targets for antibody therapy are highly needed for neuroblastoma patients with GD2-negative/low variants. One example of an additional immunotherapeutic target currently under investigation that may be of interest in neuroblastoma is the B7-H3 molecule. This member of the B7 family of immunomodulatory regulators is homogeneously expressed in both primary and metastatic neuroblastomas, as well as in a large variety of solid cancers, while it shows low or null protein surface expression in most normal tissues [54,55]. More specifically, a recent study found neuroblastoma patients with GD2-negative/low variants to still express B7-H3 molecule in high levels, suggesting that B7-H3 might represent an optimal alternative targetable molecule for these patients in particular [56]. At least one anti-B7-H3 monoclonal antibody has already been developed, enoblituzumab, which showed potent antitumor activity by peripheral blood mononuclear cells towards B7-H3-expressing tumors [55,57] and has been recently clinically tested in a phase I trial for solid pediatric tumors, including neuroblastoma (www.clinicaltrials.gov: NCT02982941, accessed on 21 April 2021). In combination with CD47-SIRPα checkpoint blockade, this could be a feasible alternative to dinutuximab for patients with GD2-negative/low neuroblastoma variants. Nevertheless, CD47-SIRPα may not be the only mechanism by which tumor cells can evade neutrophil-mediated immune destruction as neutrophils are endowed with other potent inhibitory receptors [31].

## 5. Conclusions

Collectively, our findings provide a rational basis for the combination of the therapeutic antibody dinutuximab with CD47-SIRPα checkpoint blockade to potentiate the antitumor efficacy of neutrophils towards neuroblastomas, at least of adrenergic phenotype, which is expected to significantly improve the dinutuximab responsiveness and patients’ prognosis.

## Data Availability

Publicly available datasets were analyzed in this study. This data can be found here: https://xenabrowser.net/ (accessed on 21 February 2021), http://r2.amc.nl (accessed on 22 March 2021) and https://www.ncbi.nlm.nih.gov/geo/ (accessed on 21 April 2021).

## Supplementary Materials

The following are available online at https://www.mdpi.com/article/10.3390/cancers13174261/s1, Figure S1: CD47 expression in neuroblastoma, Figure S2: GD2 expression and ADCCs of standard neuroblastoma cell lines upon overnight stimulation of neutrophils, Figure S3: SIRPα expression on neutrophils upon different stimulation conditions and on tumor cells, Figure S4: Surface marker’s comparison between a panel of neuroblastoma cell lines and ADCC of 691B and 691T with overnight stimulated neutrophils.

## Abbreviations

ADCC	antibody-dependent cellular cytotoxicity
ADRN	adrenergic
ANOVA	analysis of variance
CD	cluster of differentiation
CPM	counts per minute
DE	differentially expressed
Dimab	dinutuximab
DMEM	Dulbecco’s modified Eagle medium
FACS	fluorescence-activated cell sorting
FCS	fetal calf serum
G-CSF	granulocyte colony-stimulating factor
GD2	disialoganglioside
GEO	gene expression omnibus
GM-CSF	granulocyte-macrophage colony-stimulating factor
GTEx	Genotype-Tissue Expression project
IFNγ	interferon-gamma
IMDM	Iscove’s modified Dulbecco’s medium
KO	knock out
MES	mesenchymal
MFI	mean fluorescence intensity
Scr	scrambles
SIRPα	signal regulatory protein alpha
TARGET	Therapeutically Applicable Research to Generate Effective Treatment program
T:E	target:effector
WT	wildtype
