# Supplementary material for: CD47-SIRPα Checkpoint Inhibition Enhances Neutrophil-Mediated Killing of Dinutuximab-Opsonized Neuroblastoma Cells
Source: Cancers (Basel). 2021 Aug 24;13(17):4261. doi: 10.3390/cancers13174261 (PMC8428220; doi:10.3390/cancers13174261)
Supplement: Supplementary file 1 [file cancers-13-04261-s001.zip › cancers-1307909-supplementary.pdf]

cancers

MDPI

CD47-SIRPα Checkpoint Inhibition Enhances Neutrophil-Mediated Killing of Dinutuximab-Opsonized Neuroblastoma Cells

Paula Martínez-Sanz, Arjan J. Hoogendijk, Paul J. J. H. Verkuijlen, Karin Schornagel, Robin van Bruggen, Timo K. van den Berg, Godelieve A. M. Tytgat, Katka Franke, Taco W. Kuijpers and Hanke L. Matlung

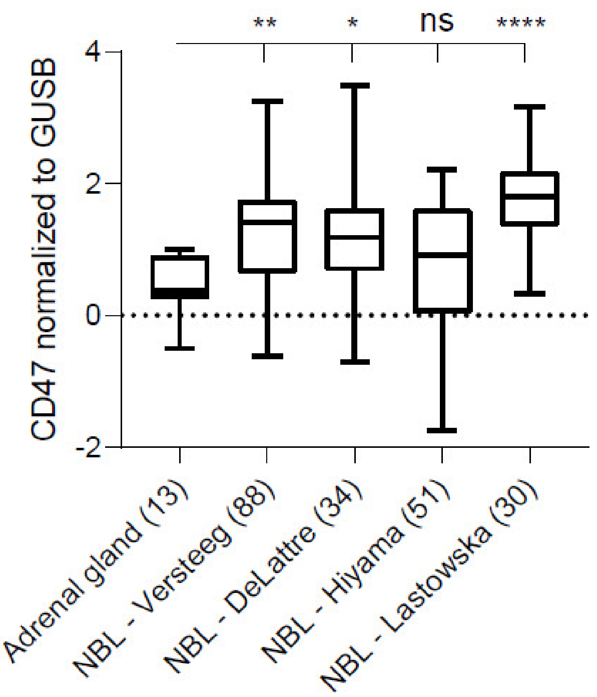

Figure S1. Normalized *CD47* mRNA expression levels in healthy adrenal gland and different neuroblastoma tumor databases. Number of individuals per dataset indicated in between brackets. Statistical significance was tested with one-way ANOVA with Sidak correction for multiple comparisons; ns, not significant; * $p < 0.05$; ** $p < 0.01$; **** $p < 0.0001$.

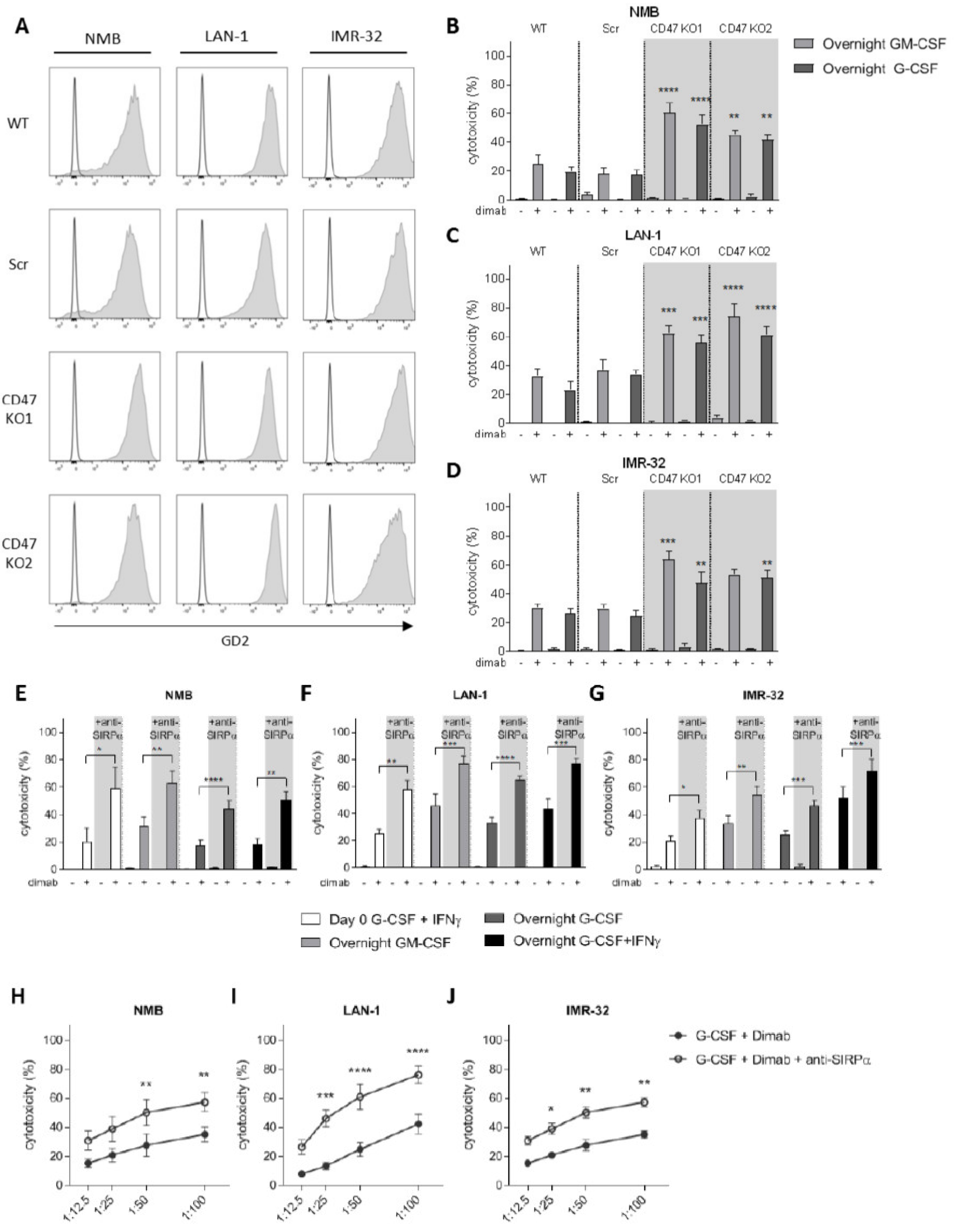

A
NMB
LAN-1
IMR-32
WT
Scr
CD47 KO1
CD47 KO2
GD2
B
NMB
C
LAN-1
D
IMR-32
WT
Scr
CD47 KO1
CD47 KO2
Overnight GM-CSF
Overnight G-CSF
cytotoxicity (%)
dimab
E
NMB
F
LAN-1
G
IMR-32
+anti-SIRPα
Day 0 G-CSF + IFNγ
Overnight GM-CSF
Overnight G-CSF
Overnight G-CSF+IFNγ
H
NMB
I
LAN-1
J
IMR-32
G-CSF + Dimab
G-CSF + Dimab + anti-SIRPα
1:12.5
1:25
1:50
1:100

Figure S2. (**A**) Representative histograms depicting GD2 surface expression ($n = 3$) as analyzed by flow cytometry on (from left to right) NMB, LAN-1 and IMR-32 control cells (top two rows) and their respective CD47 KO variants (bottom two rows). Isotype controls are represented in white. (**B–D**) ADCC of control (WT and Scr, no background) and CD47 KO (CD47 KO1 and CD47 KO2, grey background) NMB (**B**), LAN-1 (**C**) and IMR-32 (**D**) cells opsonized with (+) or without (-) dinutuximab (dimab) by neutrophils stimulated overnight with GM-CSF (light grey bars) or G-CSF (dark grey bars). n = 6–8, of 4 individual experiments. Statistics were performed by one-way ANOVA with Sidak correction for multiple comparisons. (**E–G**) ADCC of NMB (**E**), LAN-1 (**F**) and IMR-32 (**G**) cells opsonized with (+) or without (-) dinutuximab (dimab) by neutrophils stimulated with G-CSF in combination with IFNγ on day of isolation (white bars) or overnight stimulated neutrophils with GM-CSF (light grey bars), G-CSF (dark grey bars) or G-CSF in combination with IFNγ (black bars) in the absence (no background) or presence (grey background) of SIRPα blocking agent. n = 6–14, of 7 independent experiments. Statistical analysis was assessed with by a paired t-test. (**H–J**) ADCC of dinutuximab-opsonized NMB (H), LAN-1 (**I**) and IMR-32 (**J**) cells by neutrophils stimulated with G-CSF in the absence (filled circles) or presence (empty circles) of SIRPα blocking agent at different T:E ratios ranging from 1:12.5 to 1:100. n = 5, of 4 individual experiments. Statistical differences were tested with two-way ANOVA with Tukey's post hoc test; * $p < 0.05$; ** $p < 0.01$; *** $p < 0.001$; **** $p < 0.0001$. WT, wildtype. Scr, scrambled.

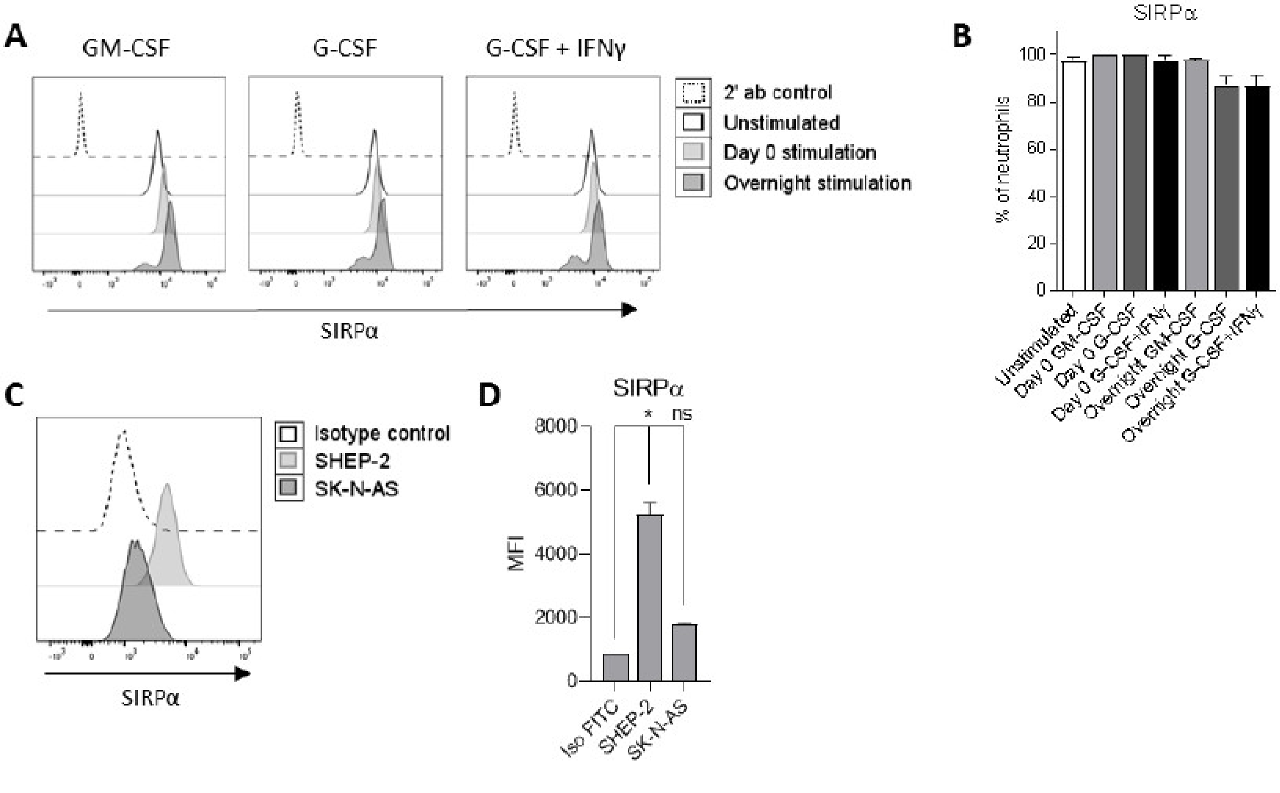

Figure S3. (**A**) Representative histograms depicting SIRPα surface expression as analyzed by flow cytometry on unstimulated neutrophils (white histograms), or neutrophils stimulated with either (from left to right) GM-CSF, G-CSF or G-CSF in combination with IFNγ on the day of isolation (day 0 stimulation, light grey histograms) or after an overnight stimulation (dark grey histograms). Secondary antibody controls are represented with a dashed line. (**B**) SIRPα expression expressed as % for the different stimulation conditions. n = 4–12, from 5 independent experiments. Statistical differences were tested with ordinary one-way ANOVA with post hoc Sidak test. (**C**) Representative histogram showing SIRPα expression as analyzed by flow cytometry on SHEP-2 (light grey histogram) and SK-N-AS (dark grey histogram) neuroblastoma cells. Isotype antibody control is represented with a dashed line. (**D**) SIRPα expression on SHEP-2 and SK-N-AS cells expressed as MFI. n = 2, from 2 independent experiments. Statistical differences were tested with ordinary one-way ANOVA with post hoc Sidak test; * $p < 0.05$. MFI, mean fluorescence intensity.

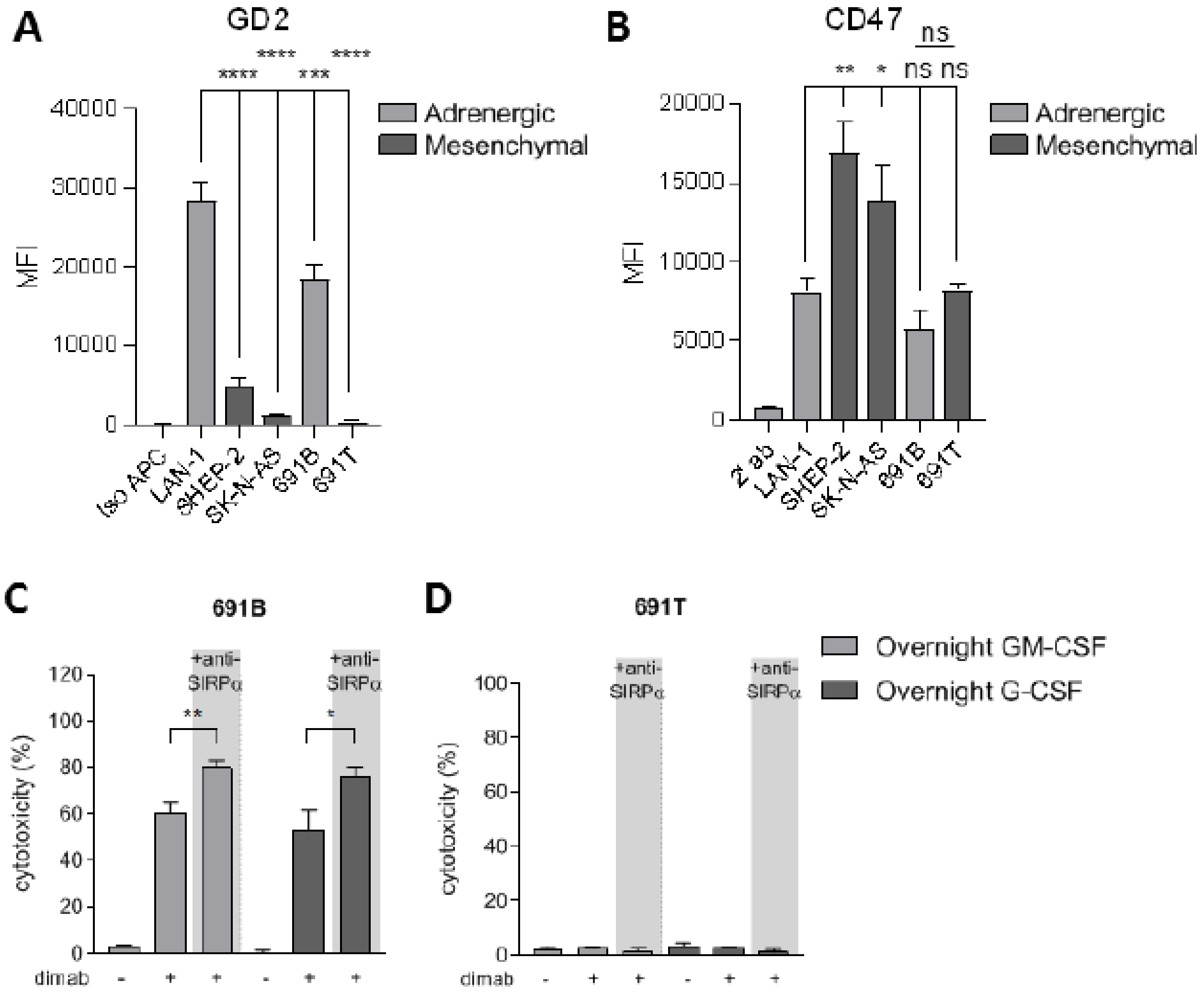

Figure S4. (**A–B**) GD2 (**A**) and CD47 (**B**) surface expression as analyzed by flow cytometry (MFI) on LAN-1, SHEP-2, SK-N-AS, 691B and 691T cell lines. *n* = 2–3, of 3 independent experiments. Statistical differences were tested with ordinary one-way ANOVA with post hoc Sidak test. (**C–D**) ADCC of primary patient-derived 691B (**C**) and 691T (**D**) cells opsonized with (+) or without (-) dinutuximab (dimab) by neutrophils stimulated overnight with GM-CSF (light grey bars) or G-CSF (dark grey bars). *n* = 4–6, of 3 individual experiments. Statistical significance was tested with a paired *t*-test; * $p < 0.05$; ** $p < 0.01$; *** $p < 0.001$; **** $p < 0.0001$. MFI, mean fluorescence intensity.
